# 2022 Using father-mediated intervention to increase responsive parental behaviors and child communication in children with autism spectrum disorder: A pilot study

**DOI:** 10.1017/cts.2018.192

**Published:** 2018-11-21

**Authors:** Michelle Flippin

**Affiliations:** University of Rhode Island

## Abstract

OBJECTIVES/SPECIFIC AIMS: Although parent involvement is recognized as an integral autism intervention component, and two-thirds of children are currently raised in 2-parent families, the majority of ASD parent research to date has focused on mother-implemented interventions, and fathers have been largely overlooked. However, fathers use interaction styles and language models that are different from mothers and may benefit children with ASD in unique ways. Thus there is a critical need in the field to expand our understanding of the potential contributions of various caregivers to communication outcomes. This investigation aimed to address this void in the research literature and contribute to clinical practice by including fathers in parent-implemented intervention, and adapting parent-implemented autism intervention to fit paternal interaction and communication styles. Specifically, this pilot study investigated the effects of a father-mediated intervention on parent use of responsive verbal and play strategies. Distal effects on child communication and pre-post changes in parental stress levels were also investigated. METHODS/STUDY POPULATION: A single subject, multiple baselines across strategies experiment was conducted with one dyad (i.e., father, child with ASD). In-home father coaching sessions were delivered weekly, targeting 4 responsive strategies (i.e., follow-in comments, follow-in directives, symbolic object play, rough-tumble play). Single subject designs are particularly suitable for autism interventions, as they allow for experimental control with participants who are from heterogeneous populations (McReynolds and Kearn, 1983). Child participant was 3 years, 1 month at the start of intervention and had previously received a received community diagnosis of ASD by a psychologist. Throughout the duration of the study, the child participant attended part-day community-based day care and received 20 hours per week of Applied Behavioral Analysis intervention both in-home and community daycare, as well as occupational therapy and speech-language therapy for 1 hour per week. The participating father was a biological parent who resided with the child continuously since birth. In addition, the father had no other formal parent training in communication intervention before participating. RESULTS/ANTICIPATED RESULTS: The results of the father-implemented intervention program yielded positive results for both father and child participant. The father quickly achieved a high level of competency using 3 of the 4 targeted strategies (i.e., follow-in comments, follow-in directives, and rough-and-tumble/physical play). Follow-in comments were used more frequently than follow-in directives and rough-and-tumble play strategies were used more frequently than symbolic play. Child use of single words increased over baseline and beginning use of multiword utterances was documented. Pre-post changes in parental stress for participating father and his spouse were not significant, however patterns of change across Parental Stress Index subscales scores were noted. DISCUSSION/SIGNIFICANCE OF IMPACT: This pilot investigation provided information regarding the treatment efficacy of a clinically relevant instructional program designed to enhance fathers’ ability to use responsive strategies to increase communicative acts or children with ASD. The results of this investigation advance clinical practice in the ASD field by providing intervention data relating to the efficacy of father-implemented instructional programs on child communication goals.

